# Discussions

**Published:** 1874-04

**Authors:** 


### Proceedings of Societies.

### DISCUSSIONS.

*Before the Eighth Annual Meeting of the Ohio State Dental Society, Dec. 4—6, 1873.*

[CONTINUED FROM PAGE 126.]

"How much is the process of Filling Teeth due to mere manipulation?."

Dr. Taft: I do not know whether I understand Dr. Way in his paper just read, but I understand him to say that success in filling teeth is dependent wholly on manipulation. Now, I regret very much if that is to be received as a truism. I should regret very much if our younger members should adopt that as a motto, and simply learn to be mere manipulators, and then imagine that they had attained all the elements of success. I think he should reconsider that statement. It is calculated to lead some astray. What constitutes success in filling a tooth? It is not merely to fill, to seal up hermetically a cavity in a tooth; that is not all; success in filling teeth includes both the manipulation and the knowledge of the treatment involved, or what is required in every case. It includes not only the filling or sealing up the cavity to the exclusion of every agent that will produce decay, but treatment that will bring about such a condition as will secure the permanency of the operation. Every reflecting person will see at once that there are many cases so involved in disease that the very best filling, so far as manipulation is concerned, will utterly fail to save the teeth. Why is it that some teeth that are filled well so far as mere manipulation is concerned fail in a very short time? Because of unfavorable conditions that attach to the case. So that more is required to secure success, to secure the preservation of the teeth, than mere manipulation, however perfect that may be. It includes a knowledge of all the conditions—a knowledge that shall enable the dentist to modify and control their conditions and establish the best possible state of health. Every thing should be done to improve the general condition; and the teeth will thereby be improved.

These things are elements that enter into the successful practice of dentistry. They are necessary in order to secure the best success. It is true sometimes, that a tooth may be filled or a particular cavity filled, and though unfavorable conditions remain in force be successful. That is a matter which often comes within the observation of the dentist. It seems to me that he who takes a case surrounded by manifestly bad conditions and merely fills up the cavity, falls very far short of the performance of his whole duty to his patient. This expression, a "perfect filling" is rather an indefinite. one. An operation in the technical use of the term, could not ordinarily be called perfect; but we are in the habit of thus speaking of these operations that seal up a cavity to the exclusion of all substances that produce further decay. A perfect filling restores the defective tooth to its original form and usefulness, nevertheless sometimes decay will take place when the cavity is as thoroughly filled as possible, and when the border of the cavity is apparently secure, still sometimes decay will occur. But upon the examination of sections of the dentine and enamel, even the best I have ever examined by the microscope, as I have shown before this body a year ago, present throughout the dentine imperfect tracks or portions that cannot perhaps be recognized by the unaided vision. Those imperfections are shown under the microscope, definitely and distinctly. These imperfections oftentimes exist upon the crowns of the tooth in the enamel where there is no depression and in them decay will take place upon a molar or cuspid. They exist in the best teeth and indeed oftentimes into and through the dentine. They would not be detected by the eye alone. And when manipulation has been thorough and the operation as compelte as possible under the circumstauces, decay may ensue.

Dr. Rehwinkle: The progress which dentistry has made in the last ten years has been great. We have arrived at the stage now where operators as well as the public expect impossible things. And I believe among operators in certain local districts a state of affairs has been brought about when the dentist has the greatest anxiety to outdo his neighbor. Teeth are filled with gold, which if a little judgment were used, would not be. I have done so, and I am satisfied others have. And you rely entirely upon your own ability to fill them to your own and everybody's else satisfaction. As far as that goes all is well; you forget that it is your intent to make an operation which shall be of permanent benefit. As far as your filling goes, it is all well, but now comes the test of time. How long have you reason to suppose that tooth will last? Will a success of two years satisfy you? Will three, four, or five? When you have spent five or six hours over the operation, subjected the patient to the pain of the operation, and all that, I say is a success of four or five years, equivalent to the expense incurred, and to the time used?

As a general principle I endeavor to show and expose as little gold as possible. Whenever I find a good bicuspid that is sound to all appearance, I leave it, saving the natural tooth as long as I can When you come to build up, if you have laid your foundation well, then building up ceases to be a matter of skill, and becomes simply a matter of time and patience. I hold that it is not good practice to fill all teeth indiscriminately just of that one material. I might state my position in two or three words: The success of dental operations depends not altogether on the mechanical part; but he who discriminates best in cases where he shall fill teeth with gold or tin, permanently or temporarily, in fact he who is able to recognize the accompanying condition of the patient will produce the best results. There is no doubt that a great many fillings are passed off, and are honestly believed to be perfect by the operators. And when you hear the teaching of men of authority, of using one peculiar kind of gold which in another case is better, it is best to examine and discriminate. A great many teeth are lost because through the adhesiveness of gold they fail to make a proper junction of the gold and the wall of the tooth. The filling is to all appearance nice, still there is through the material and through the want of appreciation of the peculiarities of the case a failure.

But notwithstandiag all, we must take it for granted that no mechanical fillings are in themselves perfect. I think that it behooves us at this present time to take a stand and make observations and see whether we are going in the true course; or whether it is best to deviate somewhat. It cannot be denied that root fillings are the rule now, while ten years ago they were the exception. Our expectations are raised too high; we are surprised when we talk about filling a dead tooth, to find an inflammation and abscess. We ought not to be surprised. I sometimes doubt whether it is merely a pathological condition.

Dr. J. Taft: We should in all cases avoid destroying the pulp of a tooth. The use of arsenic in a tooth is almost always equivalent to the destruction of a tooth. After devitalization of a tooth, it becomes more or less rapidly deteriorated, and by and by is usually disintegrated. It is all important to save alive the pulps of teeth.

3*d Subject.*—"Observations on the methods of operating, and materials used in filling teeth."

Dr. Herriot: This subject appears to me to be intimately connected with the second subject. The methods of operating are varied, as we all know. Some use excavators, some use chisels; all the different instruments are used to operate in filling; also there are various modes of applying material. Some use hand pressure for the purpose of packing gold in the cavities. There seems to be no end to instruments for the preparation of cavities. The buring engine is used by many with a two-fold injury. One is cutting away too much of the tooth by careless handling; another cuts into the pulp cavities; it is used by some to cut away the natural tooth. I feel that it is the duty of the dentist to do all within his power to save the natural teeth even although he may find it necessary to make great efforts.

We find that the mode adopted by some in using a hand glass is a decided advantage; good operations can be performed where we cannot see the operations without the aid of the glass.

Dr. Warner: I want to say a word or two about amalgam. I have always used more or less and for several years have made my own. I think we ought to know what we are using when we use amalgam. For some years I used five parts tin and four of silver. Finally I have been using five parts of tin and seven of silver. I have recently learned that Dr. Knowlton is making his own amalgam and using less silver than I. In making a cast take the tin, alloy it with silver and stiffen the tin so as to make it suitable. I think good amalgam can be made with about as much silver as I use. Dr. Knowlton would use bismuth.

Dr. Clippinger: My observation and that of those who are recognized as good operators is, that with amalgam it is but a short time before the filling is undermined. While the patient thinks the teeth are being preserved by amalgam, it is often found that their destruction is going on. I have also seen gold filling undermined, but it is not very common. It is the exception. There is difficulty in recommending the use of the base metals The cost has a demoralizing influence upon the public. There is no use to spend skill upon a tooth, introducing a material that will deceive the patient and fail ultimately to preserve the tooth.

Dr. Berry spoke in favor of the use of amalgam in filling. His experience as well as observation shows that teeth filled with amalgam do not become devitalized and are permanently saved.

9*th Subject.*—"Does the administration of lime phosphates aid in the development of good teeth?"

Dr. Herriott: There is great importance in people's looking to it that their children may have good bones and healthy teeth. A woman came to my office one day to have her teeth filled. She was operating in a book agency. The book was a book instructing mothers how to live during pregnancy. I at once condemned the book as well as herself. She became enraged at my suggestion. She insisted that all dentists were a set of scoundrels. We used to feel the importance of our profession and our position before the public in this very particular: to see that they are furnished with that food which i necessary for health and sustenance. Many give out because their frame-work is not good. I do not believe that it is necessary to give heroic doses. It is not in accordance with my practice.

Dr. Porter: Some gentlemen said, "Talk about arsenic being a tonic." I did not say so. It is not material, however, but if these gentlemen lived in a malarious district they would give arsenic. As far as the heroic treatment is concerned, locality affects that also. Some one has remarked that the dentists are in the habit of following a certain routine. For the last eighteen years essay after essay has been written on the subject of modifying the condition of the teeth, and the recommendation has been, year in and year out, Give them phosphate; and that is all there is of it. You take the practitioner of medicine, and five cases out of six he paves the way for the reception of the very disease that he expects to relieve. You must have the general system in such a condition as to bring about the assimilation of these elements. Now, if there is not anything in modifying but the simple administration of the phosphate, then it is a very easy thing to do. I have tried to produce these results in certain cases without any preliminary treatment. I failed. It may have been on account of the malarious district in which I live. However, these are the results, and of course the doses that you give will materially depend on the locality in which you reside. It is time, perhaps, that we should do a little thinking for ourselves, and a little earnest experimenting. If we would do that we would not have to depend on books and formulas so much as we do.

With regard to wheat procured from lands with which I am acquainted, I believe I derived a benefit therefrom. We sometimes assume that wheat is wheat; but I believe there is a difference—evidently a difference in the constituent elements of the different kinds. Some have a heavier covering, which is the part that contains more phosphate than others. I have had the pleasure of observing the effect of this kind of treatment in the rearing of children. I have seen a child born with soft, gelatinous bones. Such have very imperfect skulls.

Then again, I have seen children with skulls almost perfect, teeth hard and solid, at the age of five years, with not a speck of decay, perfect and beautiful—simply as the result of proper hygienic treatment, the mother living in harmony with the conditions in which she was placed, and the child being fed with proper kind of food, a proper hygienic regimen being observed. There is a great deal in this subject, and the duty is upon us as professional men to educate in this direction.

Dr. J· Taft: I am very sorry that I have not been able to be here during the discussion. My ignorance of what has been said has cut me off from any extended remarks on the subject. It is a very interesting subject, but it seems to me that two or three, who have referred to the matter within a few moments, only take a partial view, or see the subject in a particular aspect. It is a subject in which much is involved. The mere feeding of such food as pabulum will not accomplish much unless the assimilating and appropriating power is effective. There are many kinds of food that are rich in the required pabulum. There are certain conditions that are necessary in order to enable the system to properly appropriate. Now, food of any kind should be properly prepared in all respects when taken into the body. The processes in the nutrient function should be as perfect as possible, at every step, from the reception of the food until the material is built up in the tissue. The ability of the system to take and appropriate any pabulum will be modified, of course, by a great variety of circumstances. Doctor Butler was relying largely on fresh air; but there are other things just as important. There are very many things that exercise an influence in this direction. Shut up in a dark room, there would be difficulty for want of sun-light, though the air might be good. Also the influence of the mental conditions on the physical, that must be taken into account. The mind is a support to this body, and is largely instrumental in the appropriation of pabulum that is necessary for support. Consequently these mental conditions must be taken into account. Frequently the mental condition is such as to poison that which is taken as pabulum. How often is it that the excessive mental disturbance will poison the mother's milk—nature's preparation—and the babe will sicken and die! And under such circumstances the mother suffers too. All these things should be taken into account. Quite as much depends upon the mental condition of the mother as anything else for the child's well-being. Life is many a time destroyed by excessive mental disturbance. We are liable to overlook these things in our study of hygiene. "Will the system appropriate more than a certain amount of what may be given it?" Is not this question the result of a proper and full conception of the whole range of the subject? It is true you may overfeed, but very seldom will any excessive appropriation be made. If there is material enough brought to hand to supply the demand, there will be defect, of course; and there may be material enough brought to the workmen, and the builders so demoralized, so enfeebled, as not to be able to build it up into the proper structure.

*4th Subject.*—"Why has not dentistry received the general recognition as a specialty of medicine to which it is entitled?"

Dr. Warner: I do not know that I have much to say on this subject, but I will say what I have in few words. For several years it has been claimed that we are a profession. It may be that we are young in years; but, if success is what we are to be gauged by, I claim that the profession of dentistry is as old as the profession of medicine, and is certainly as old as the profession of surgery I will make just one or two points here, and that will be to claim, that much that is great and grand in surgery to-day is derived from the knowledge that the profession of dentistry has taught the surgeon. Now, what would surgery be, were it not for anæsthesia—chloroform and ether? There is just one point here, and I will make it from the annals of the army record of the last war. I refer to the late rebellion. Now, surgical experience in the hospitals of the Southern armies, where they were deprived of ether and chloroform, shows that there was a death-rate of about seventy or eighty per cent. In the Northern armies, where we had access to these agents, the death-rate was reduced to eighteen per cent. This great difference in percentage is due, it is claimed, to their presence and use. These things, if the dental profession had no more, would be sufficient to establish its claim to recognition as a specialty of medicine.

Dr. J. Taft: "Why has not dentistry received the recognition it is entitled to?" My impression is that, under the existing circumstances, it has received all the recognition to which it is entitled. Now, if this question conveys the idea that dentists do not receive all the recognition or the kind of recognition they should have, we should consider, Is it true? There are two things to be considered in this matter: First, Do dentists place themselves in the position they ought to occupy? Do they make themselves transparent, so that everybody can see them in their true light? Are we not at fault in this matter? Do we not present something else than our true professional status? Transparent people are rare; we love to point to the friend of whom we can say, "I can see through and understand him; I know him." The other kind, whom we cannot see through, we think of in a different light; we do not admire them. Do we make such presentation as professional men we should? Present such professional status as ought to entitle us to the position we claim? If we are not recognized as we desire, whose fault is it? Simply our own. If I endeavor to make A, B and C believe that I am something that I am not, I am a living lie, calculated to deceive everybody that looks at me. Are we not too often presenting ourselves in another character than we should assume to hold? We should also look at the ability of those who criticise. Many, of course, cannot see us in our true light. The blindness and ignorance of others are elements to be taken into account here. If, then, it is true that the dentists do not have their position recognized as they ought to be, there are two reasons for it: one is, that they do not present themselves in their true light; the other is, the blindness and inability of people to see.

What shall we do? Mourn and lament over it? It is beneath a proper dignity. The dentist does occupy a high and honorable position. We should march right along in the line of duty, performing duty to the full, and then our honor will take care of itself. All that we really deserve we will gain. We ought to be able to rise above these petty matters of criticism. The man who thus takes circumstances and makes them tributary to himself will care very little about what people say of his abilities, or of their neglect to appreciate him. Duty is the matter. We ought to examine ourselves and endeavor to deserve the appreciation of all.

Dr. H. A. Smith: The reason we are not recognized as specialists is, because we have detached ourselves from medical men. When we do not separate ourselves from choice from them, they will recognize us. When we go through the same course of study that the medical student does, then we will be recognized It is indispensable to go back to the foundation, as others have. If we are educated as thoroughly as our physicians are, they would have to recognize us.

Dr. J. Taft: I protest against the admission that the dental profession do not study the subjects which are studied by the ordinary medical student. We do study the foundation principles.

Dr. Smith: I mean by the admission that we do not pursue the study of medicine to the extent that they do. I repeat that we must be educated as they are before we will be recognized as we desire to be.

6*th Subject.*—"What are the best materials for filling roots of teeth?"

Dr. Siddall: I have used hickory wood for filling roots. I had a case of two bicuspids that had been filled twenty years, by Dr. Robinson, of Cleveland. One had been discharging seventeen years. I removed the gold fillings, and as preparatory treatment cleaned out the canals of these bicuspids and drove a piece of hickory into the canals, filling them temporarily, designing to treat them finally. They remain now as they were. In consequence, I have used some hickory for several years. I have been very greatly delighted with the result. It can be done very well by saturating the piece of wood with carbolic acid.

Dr. I. Williams; The first point is, to close the apex, so that no extraneous matter can accumulate. I do not believe that hickory wood will do that perfectly. I got some ideas from the Dental Register, perhaps a year ago, upon that subject, that seemed to me to be very sensible, and I have been working on that system; that is, of filling the apex and canal with tin foil. After seeing the statement in the Dental Register, I took teeth, extracted, teeth and made instruments as there suggested, filled them in my laboratory, and split them open to test the perfection of the work; and was gratified with the perfect manner in which I could consolidate tin foil in the nerve canal, and the perfection with which I could carry it to every part of the tooth. When I use tin foil for the purpose of filling the roots of teeth, the tin foil becomes at once perfect. It can be carried to any point almost.

Now I have a little experience in my own mouth, that would lead me to object to the method of Dr. Siddall. I have a tooth in my mouth, the root of which was filled with gold wire; and I have had trouble from the fact that the apex of that root is not filled, neither is the nerve canal perfectly filled, as no hickory wood could adapt itself to the nerve canal perfectly. The apex being open, difficulty ensues as the result. I do not believe that would or could have occurred if the apex of that tooth had been perfectly filled, even if the nerve canal had not been filled at all. But I use tin foil, say half way, and then commence with gold. We know that when two metals come in contact in the mouth, different metals above and below the tongue, there will be an electric current; but, in the nerve canal, where no fluids can reach the metals, I think there will be no galvanic action, and, consequently, I have not been careful at all to introduce a metalic substance between the tin and the gold. I have never to my knowledge had any recurrence of abscess after careful treatment.

Dr. Butler: I like to be correct and correct others if I am satisfied that they are wrong. I can not endorse the modes of either of these gentlemen in filling canals, notwithstanding they may have had the success they claim. But the reason I do not like it is, I am afraid that they will have trouble which they do not anticipate. Now, I have been through all these different modes of treating the nerve canal in the teeth, and have endeavored to make careful observations of my own operations; they agree with the observations of others. What may seem to be a success in their hands may only be a success by comparison. How can he make comparison, if he does not carry his observations outside of his own practice? Does his success alone make a real success? It is only in degree. Dr. Williams fills the teeth with tin. There is a serious objection, and I think you will see it. He can have had but one difficulty up to the present time, but if he should have a similar difficulty arise which I have seen he would regret that he ever put tin in the root. He says that there can be no moisture whatever come in contact with the metal. I think he does not mean what he says. The root, you must remember, is not a dry substance, incapable of permeation by moisture, but it admits of nourishment, and must or you would have a dead body. Consequently there must be moisture or a chance, at least, for it to come in contact with the canal. What is the result? The root of the tooth of a young lady, one of my patients, I filled with tin. It showed very unpleasant complications. The root was filled with tin and the crown with gold. In the course of a year that tooth looked blue, up by the neck. When I came to examine it, I found it was owing to the oxygenation of the tin in the root. That is the reason why I would not fill canals with tin. If I am going to fill a tooth with the two metals, gold and tin, I put the gold down in the root and the tin in the crown. To use all tin or all gold wire is not good practice. I make my judgment up, because I have not and others have not used them. You can not fill with them tightly. It is impossible to do so. There is no necessity nor demand to do it in that way. I make this statement understandingly, intelligently, from observation where that kind of practice has been pursued. If you want to fill a root well, prepare it nicely for the gold, and then insert it skilfully.

Dr. Herriott: I would rather hear from our professional brethren on the subject of saving the teeth alive. It is of greater importance. My success in saving teeth where the nerves have become exposed was not very encouraging, until I adopted the practice of preparing the pulp for filling, filling the cavity with creosote and oxide of zinc, moistening it and then applying it to the nerve. Put that over the pulp and then press down gently with a piece of soft spunk, which will absorb the creosote. After this, apply the chloride of zinc, and, when it has become sufficiently solid, apply the metal filling. This, I find, obviates what sometimes follows where the chloride of zinc alone is used.

